# Supplementary material for: Metabolomics-Based Machine Learning for Predicting Mortality: Unveiling Multisystem Impacts on Health
Source: Int J Mol Sci. 2024 Oct 30;25(21):11636. doi: 10.3390/ijms252111636 (PMC11546733; doi:10.3390/ijms252111636)
Supplement: Supplementary file 1 [file ijms-25-11636-s001.zip › ijms-3267442-supplementary.pdf]

Supplementary Material

Table S1. XGBoost parameter set used in the mortality model.

**Hyperparameter**	**Definition**	**Value**	**Default**
learning_rate	Learning rate	0,0202	0,3
n_estimators	Number of estimators	440	100
gamma	Minimum split loss	0	0
max_depth	Maximum tree depth	5	6
min_child_weight	Minimum sum of instance weights in a child	2	1
subsample	Sub-sample ratio of training instances	0,46	1
colsample_bytree	Sub-sample ratio of columns for each tree	0,52	1
colsample_bynode	Sub-sample ratio of columns for each node	1	1
colsample_bylevel	Sub-sample ratio of columns for each level	1	1
reg_alpha	L1 regularization term	0	0
reg_lambda	L2 regularization term	1	1
scale_pos_weight	Weight balance between positive-negative class	8	1

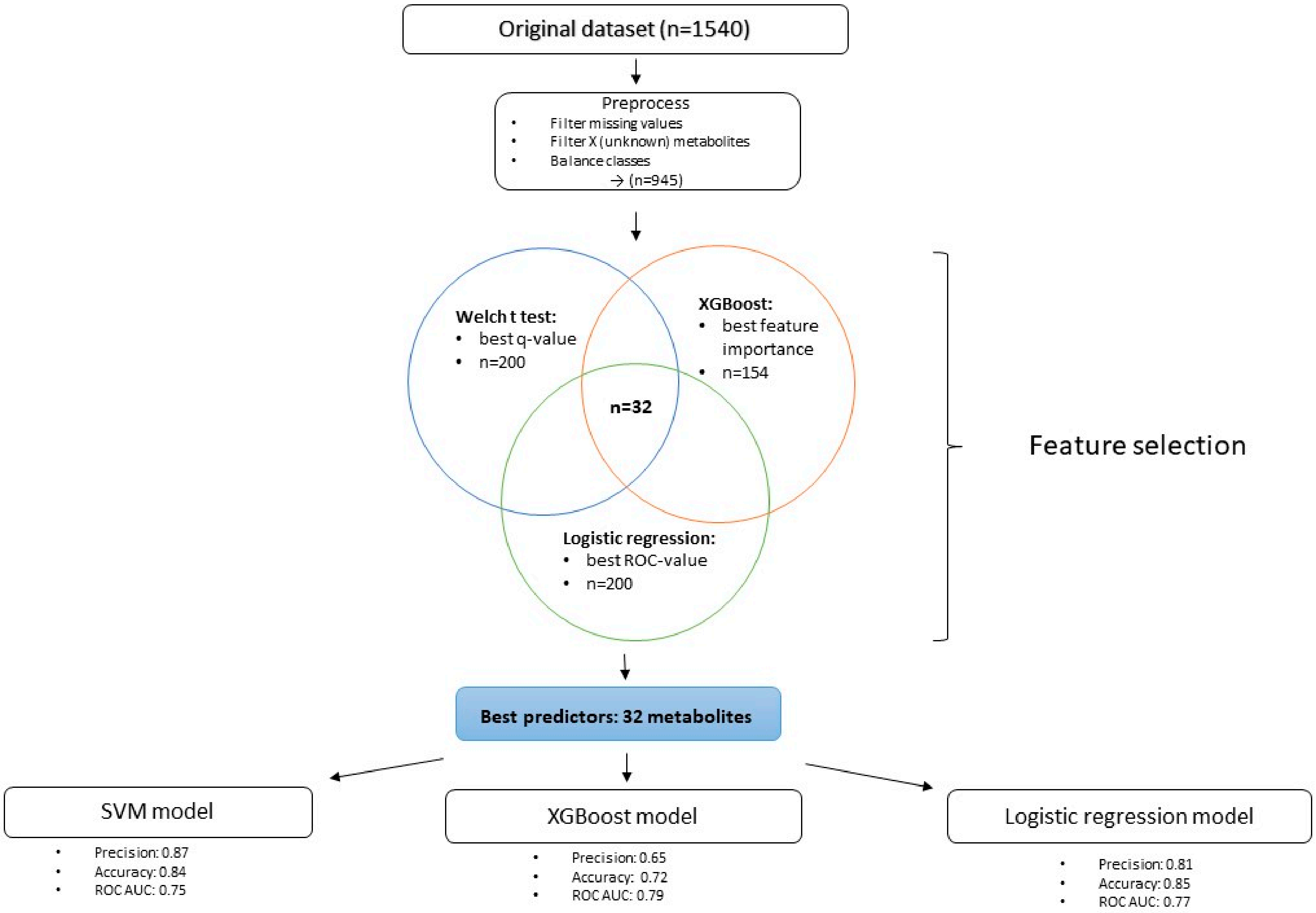

Figure S1. Processing of the data from the original dataset to the identification of the most impactful metabolites predicting mortality

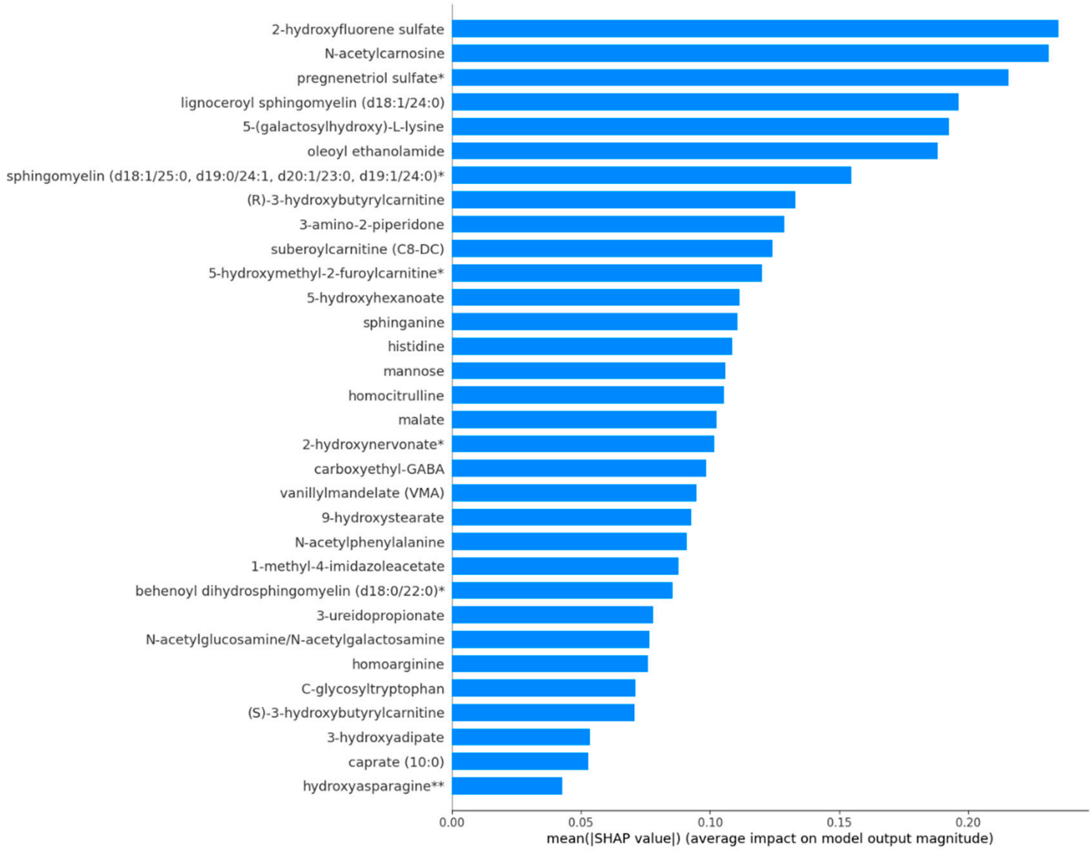

Figure S2. The relative importance of the metabolites on mortality prediction shown by absolute SHAP values.* indicates a tentatively identified metabolite; ** signify a well-characterized compound with minor identification uncertainty.

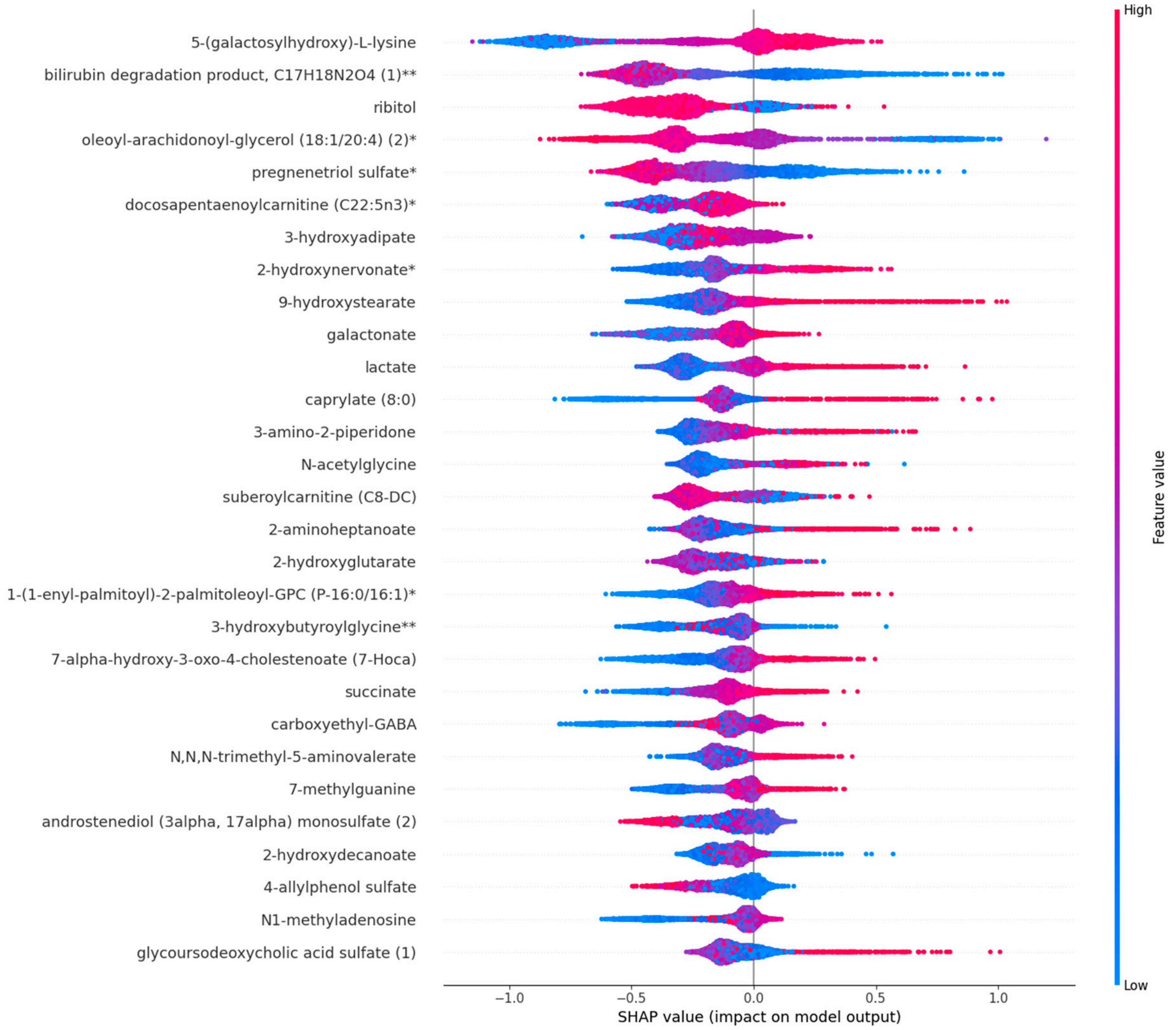

Figure S3. SHAP summary plot of the most impactful predictors of short-term mortality. Positive SHAP value indicates an increased risk prediction of mortality and negative SHAP value indicates protective effect. Each dot corresponds a single observation and the highest value of the variable is shown in red and the lowest value in blue.* indicates a tentatively identified metabolite; ** signify a well-characterized compound with minor identification uncertainty.

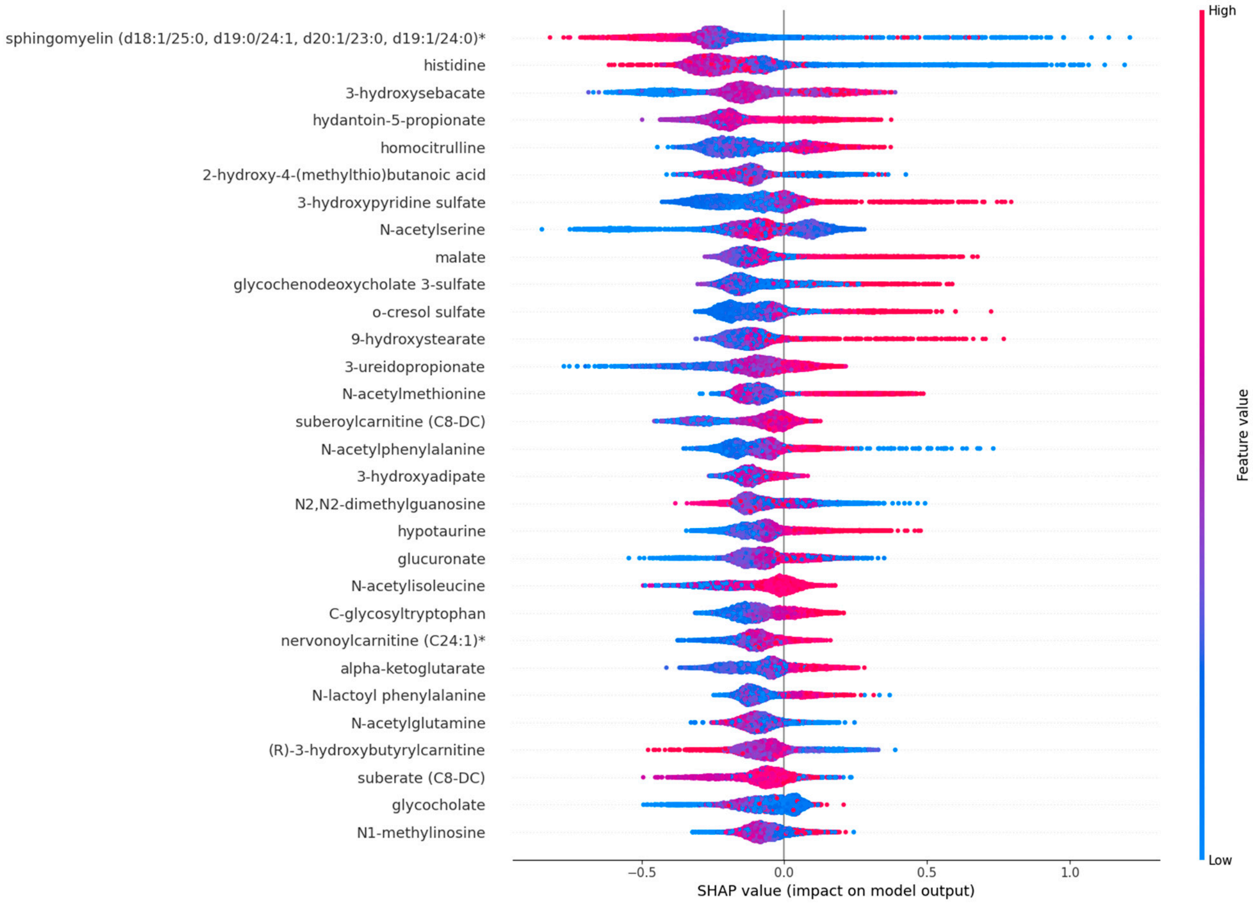

Figure S4. SHAP summary plot of the most impactful predictors of intermediate-term mortality. Positive SHAP value indicates an increased risk prediction of mortality and negative SHAP value indicates protective effect. Each dot corresponds a single observation and the highest value of the variable is shown in red and the lowest value in blue. * indicates a tentatively identified metabolite; ** signify a well-characterized compound with minor identification uncertainty.

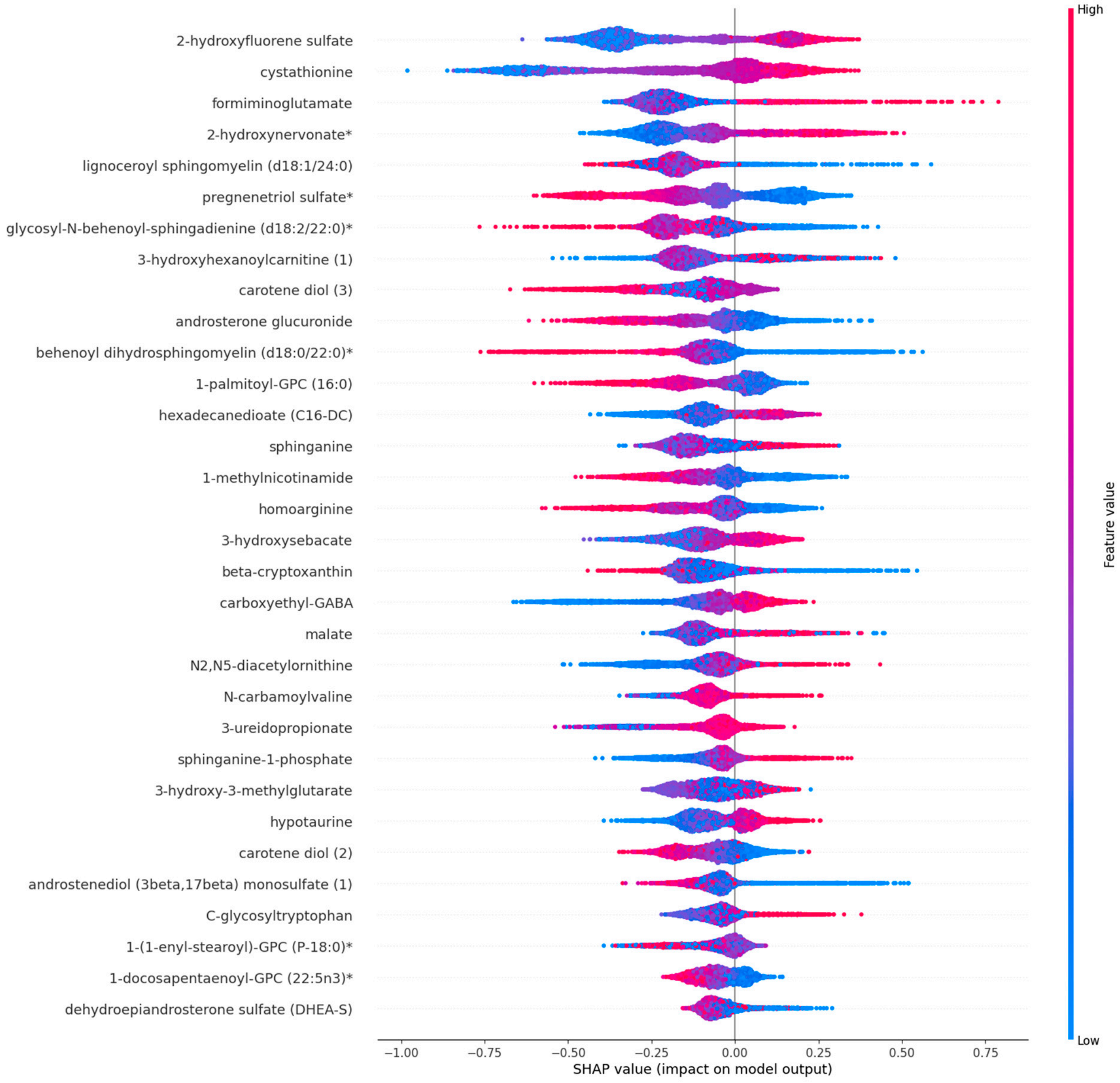

Figure S5. SHAP summary plot of the most impactful predictors of long-term mortality. Positive SHAP value indicates an increased risk prediction of mortality and negative SHAP value indicates protective effect. Each dot corresponds a single observation and the highest value of the variable is shown in red and the lowest value in blue.* indicates a tentatively identified metabolite; ** signify a well-characterized compound with minor identification uncertainty.

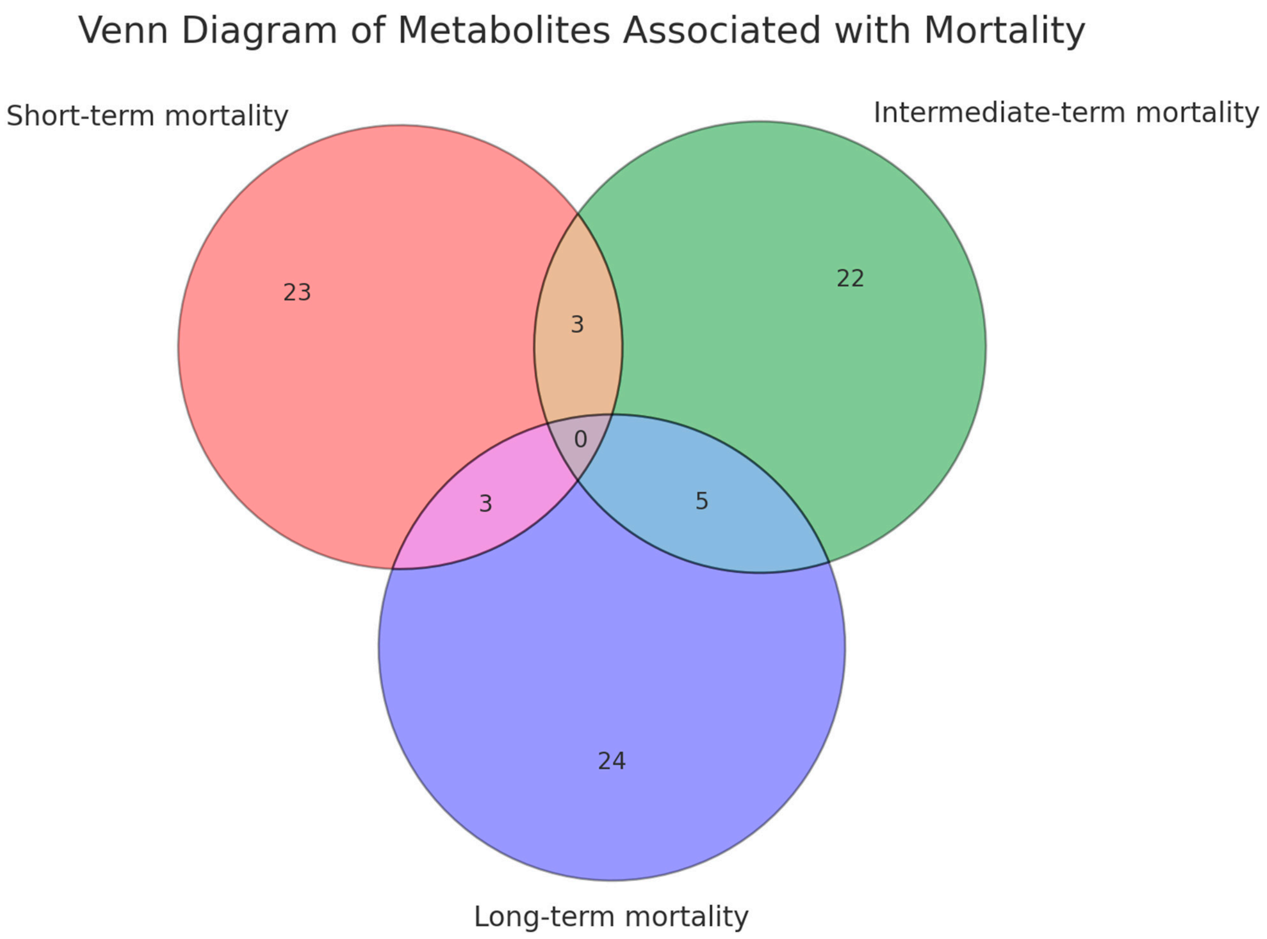

Figure S6. Distinct and overlapping metabolites associated with short-, intermediate-, and long-term mortality.

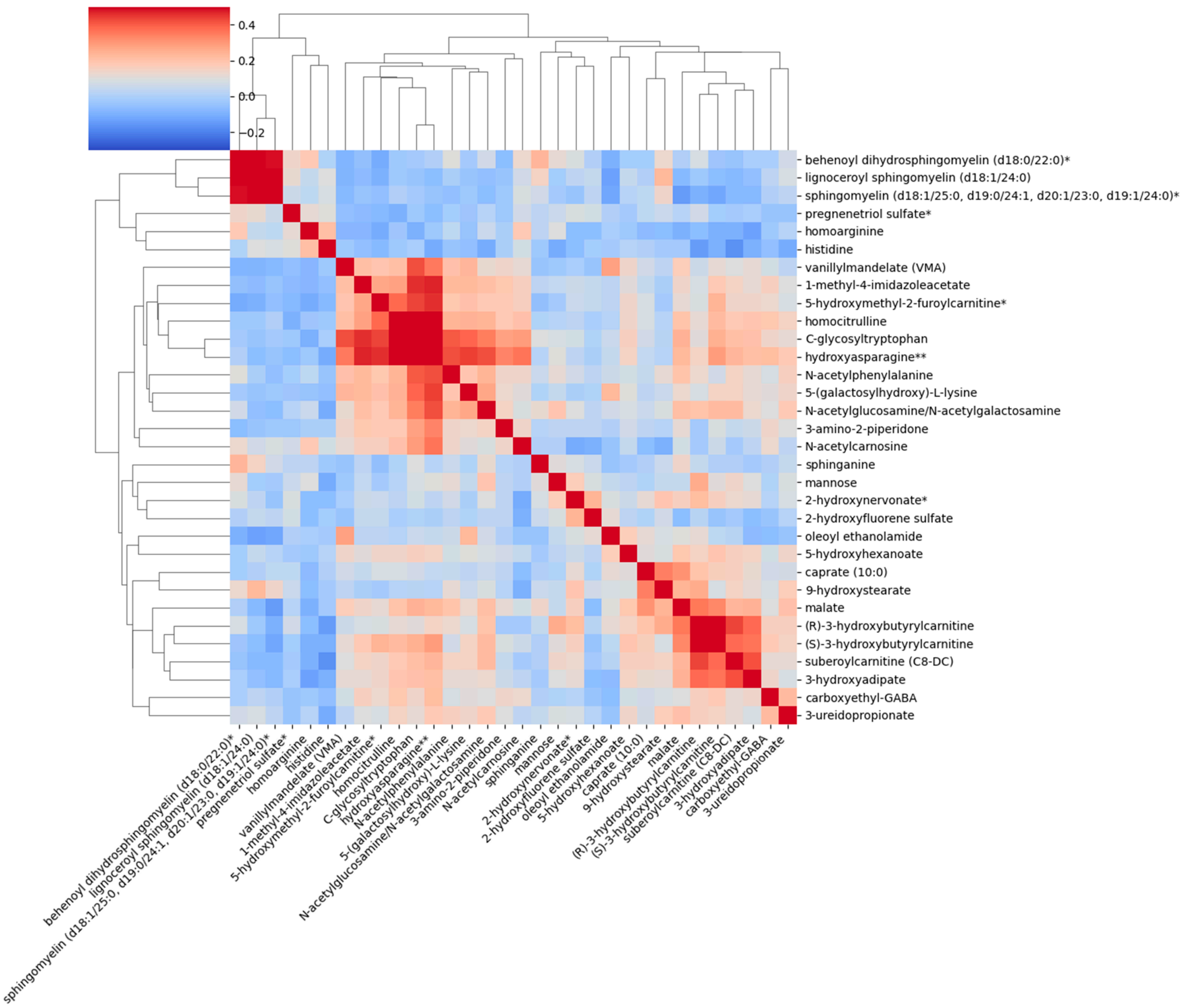

Figure S7. Clustered heatmap of 32 metabolites. * indicates a tentatively identified metabolite; ** signify a well-characterized compound with minor identification uncertainty.

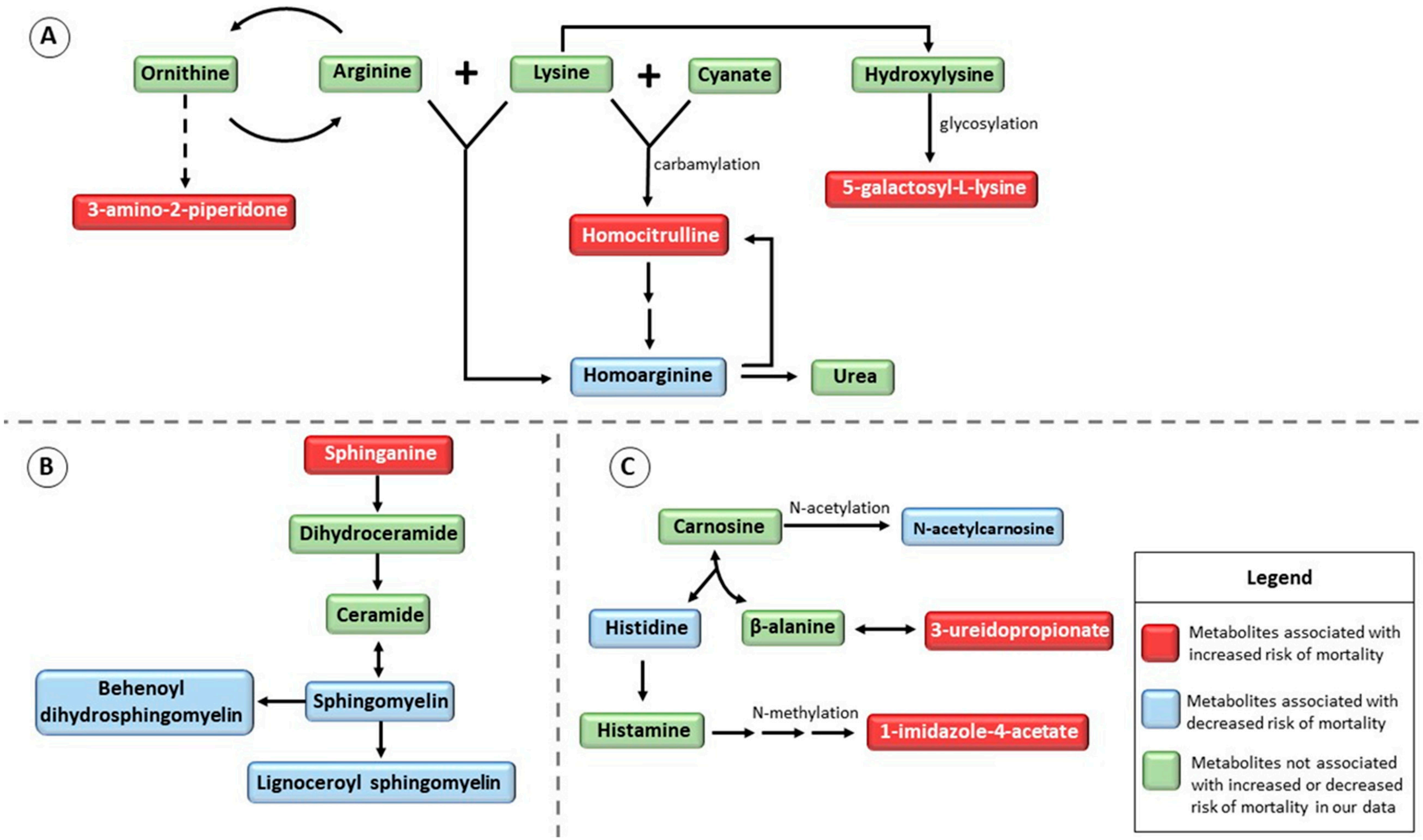

Figure S8. Main metabolic pathways of the metabolites associated with increased or decreased risk of mortality. **A**. Disruptions in the ornithine cycle result in an increased abundance of 3-amino-2-piperidone, while cyanate-induced carbamylation generates homocitrulline from lysine. **B**. Sphinganine, a ceramide precursor, generating sphingomyelin, behenoyl-dihydrosphingomyelin and lignoceroyl sphingomyelin. C. N-acetylcarnosine and histidine are carnosine metabolites. **C**. N-acetylcarnosine and histidine are carnosine metabolites, while 1-methyl-4-imidazoleacetate is the main histamine metabolite.

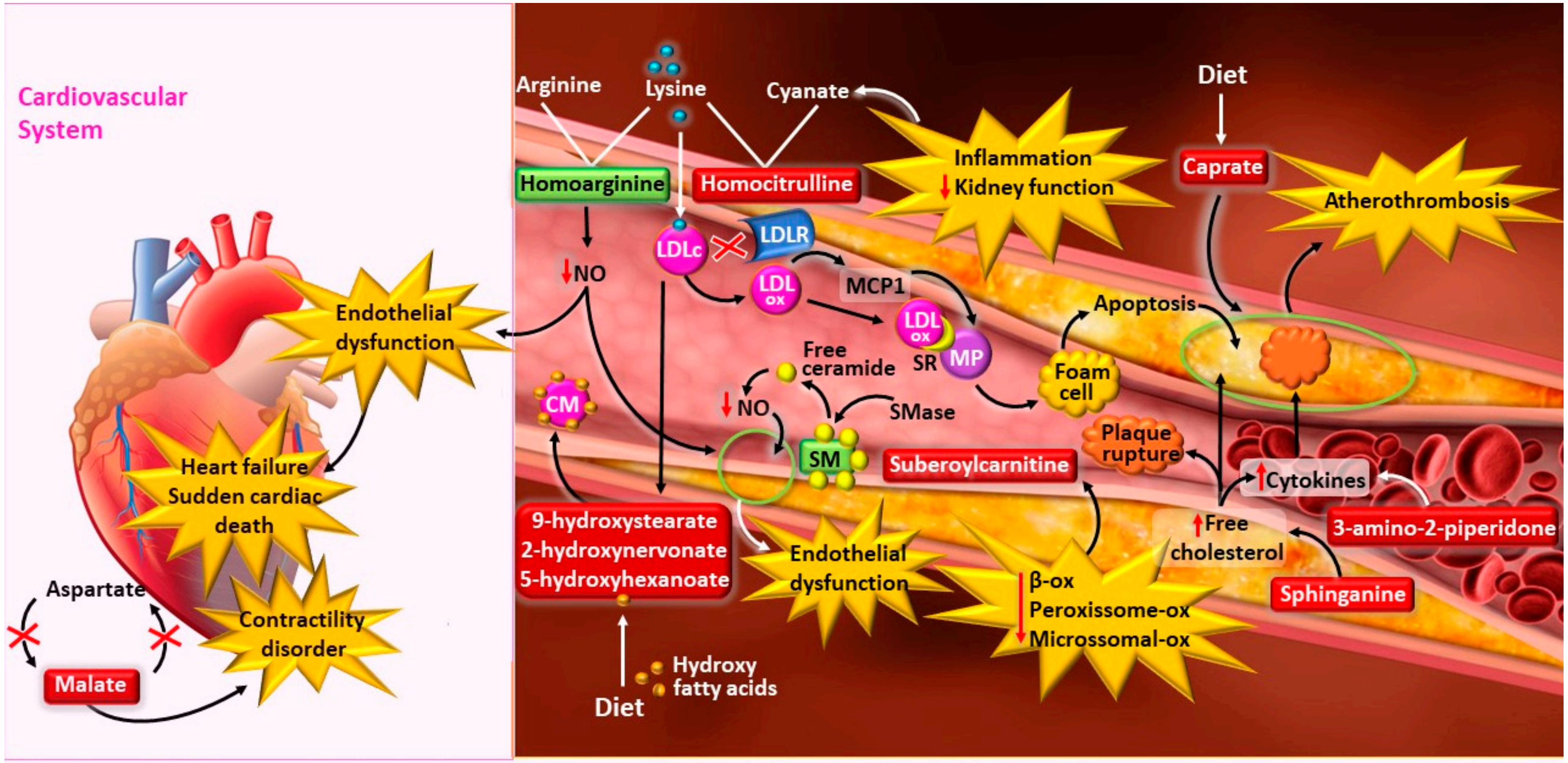

Abbreviations: CM, chylomycrons; LDLc, low density lipoprotein cholesterol; LDLox, oxidized LDL; LDLR, LDL, LDL receptor; MCP1, monocyte chemoattractant protein-1; MP, macrophage; NO, nitric oxide; ox, oxidation; SM, sphingomyelin; SMase, sphingomyelinase; SR, scavenger receptors. *Red boxes show metabolites associate with increased risk of mortality in our data. Green boxes show metabolites associated with decreased risk of mortality in our data.

Figure S9. Metabolites affecting the cardiovascular system. Distubance in the malate-aspartate cycle leads to increased malate levels and contractility disorder in the heart. Inflammation and decline in kidney function generates cyanate which carbamylates with lysine yield homocitrulline. Lysine might be directed to the homocitrulline pathway, decreasing the generation of homoarginine, and consequently of NO, resulting in endothelial dysfunction, which can result in heart failure. Lysine from LDLC can be carbamylated to generate homocitrulline, and oxidized LDLC, which is unable to bind LDLR, and in turn accumulates attracting macrophages, and generates foam cells, contributing to the atherosclerotic plaque formation. Caprate also contributes to atherosclerotic plaque. Hydroxy fatty acids coming from the diet are incorporated into

chylomicrons. Degradation of sphingomyelin generates free ceramides, which decrease NO levels, causing endothelial dysfunction. Disruption of β-oxidation, peroxisome and microsomal oxidations leads to an increase in suberoylcarnitine levels. Sphinganine leads to an increase in free cholesterol and cytokines, which accumulate and contribute to plaque rupture. 3-amino-2-2piperidone leads to increase in cytokines, promoting atherothrombosis.

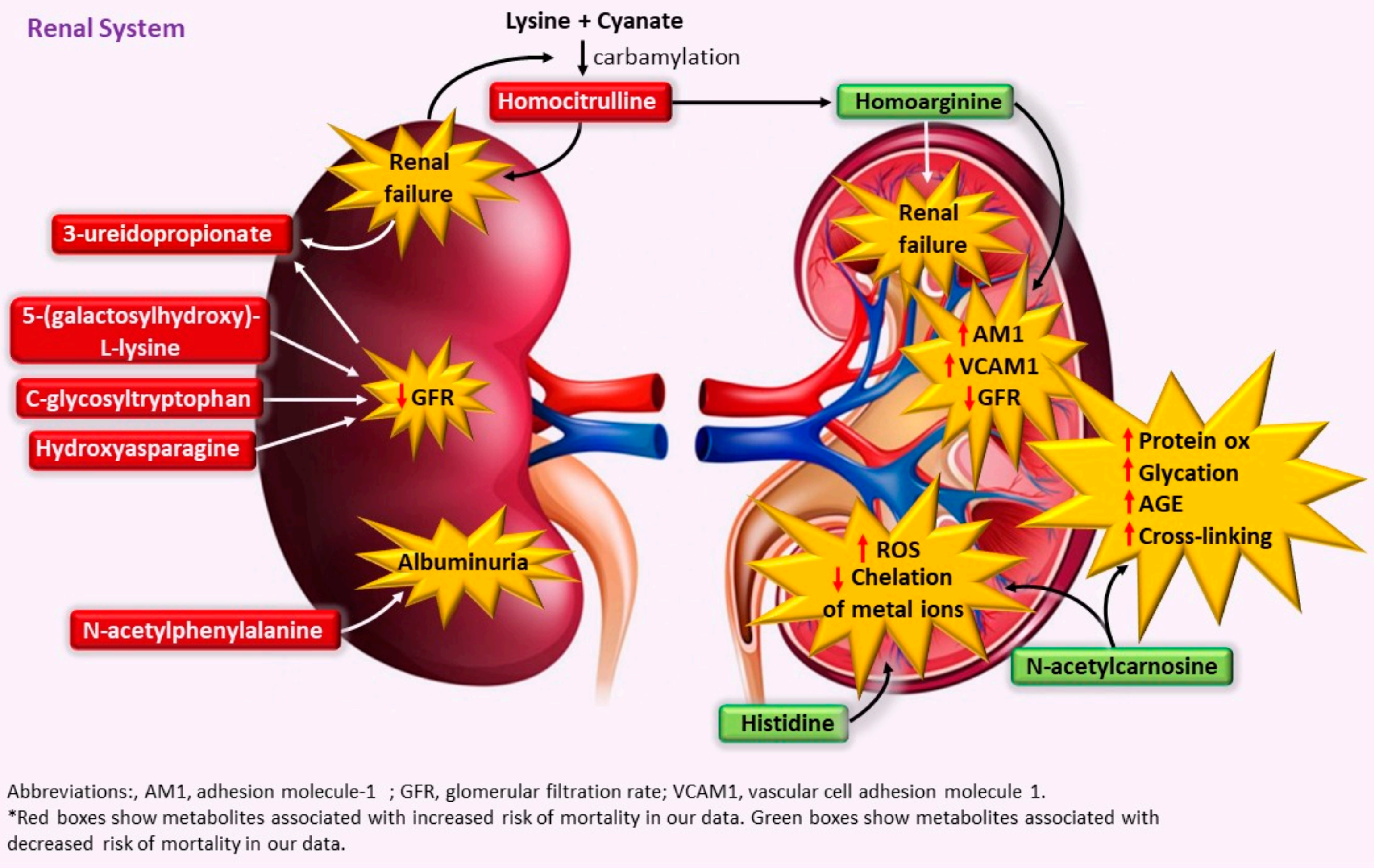

Figure S10: Metabolites affecting the renal system. Decline in kidney function leads to an increase in cyanate, which is carbamylated with lysine to generate homocitrulline, which is associated with renal failure. Progression of renal failure and decrease in GFR generates 3-ureidopropionate. 5-(galactosylhydroxy)-L-lysine, C-glycosyltryptophan and hydroxyasparagine decrease GFR. N-acetylphenylalanine is linked to albuminuria. Since lysine is directed to the homocitrulline pathway, homoarginine levels are decreased, which increases AM1 and VCAM1, leading to endothelial dysfunction, a decrease in GFR, and renal failure. Low levels of N-acetylcarnosine and histidine increase ROS and decrease chelation of metal ions. N-acetylcarnosine increases protein oxidation, glycation, cross-linking and AGEs generation.

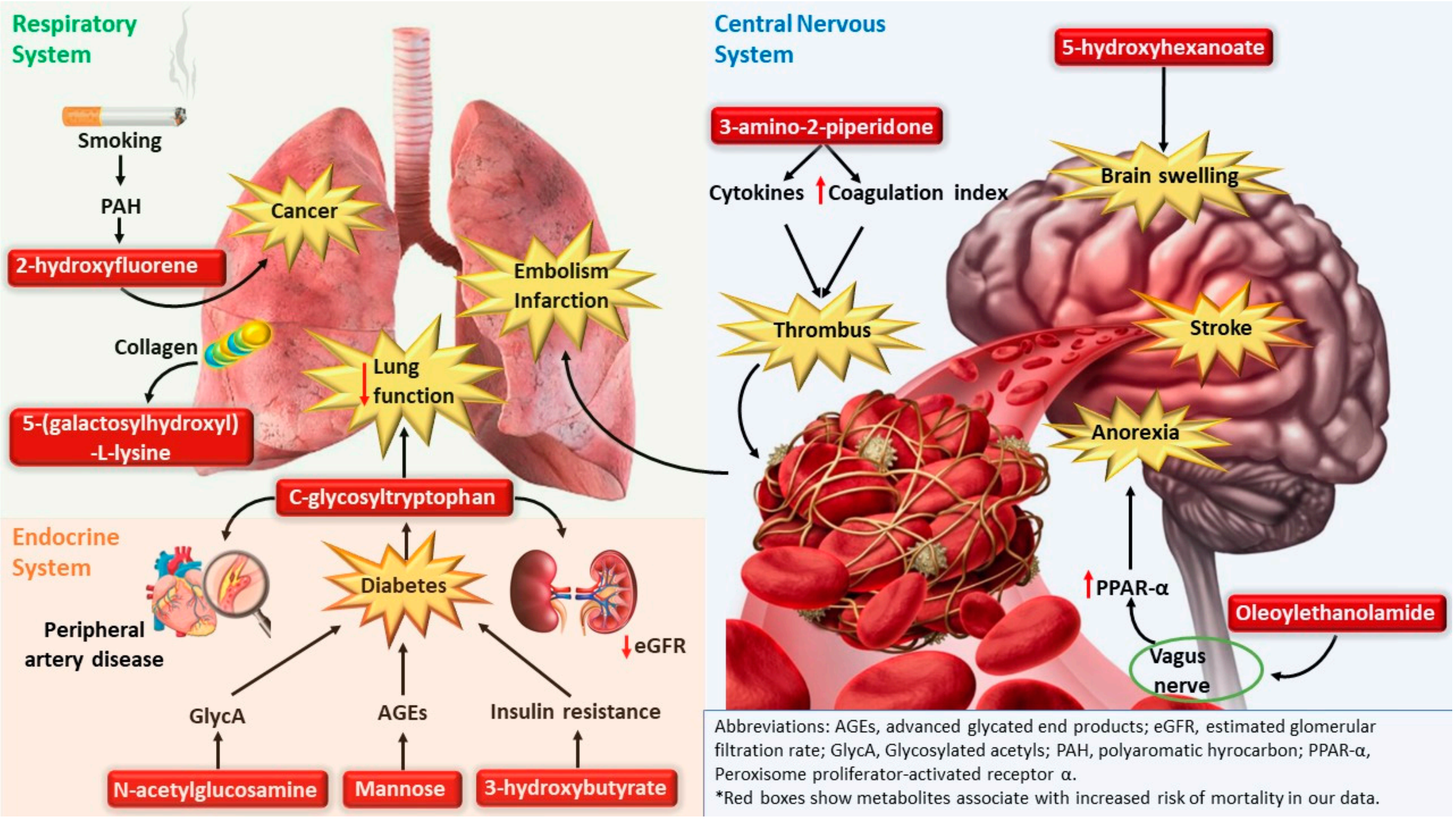

Figure S11. Metabolites affecting respiratory system, endocrine system, and central nervous system. Smoking increases PAH and consequently 2-hydroxyfluorene resulting in an increase in the risk of lung cancer. Degradation of collagen in the lungs generates 5-(galactosylhydroxy)-L-lysine. N- acetylglycosamine, mannose and 3-hydroxybutyrate contribute to the development of diabetes by generation of GlycA, AGEs and insulin resistance. Diabetes leads to an increase in C-glycosyltryptophan, which contributes to the development of peripheral artery disease and a decrease in GFR. C glycosyl tryptophan also contributes to a decrease in lung function. 3-amino-2-piperidone increases cytokines generation and coagulation index, leading to thrombus formation, that can lead to lung embolism or infarction, and cerebral stroke. Oleylethanolamide acts on the vagus nerve, increasing PPAR-α, leading to anorexia. 5-hydroxyhexanoate is associated with brain swelling.

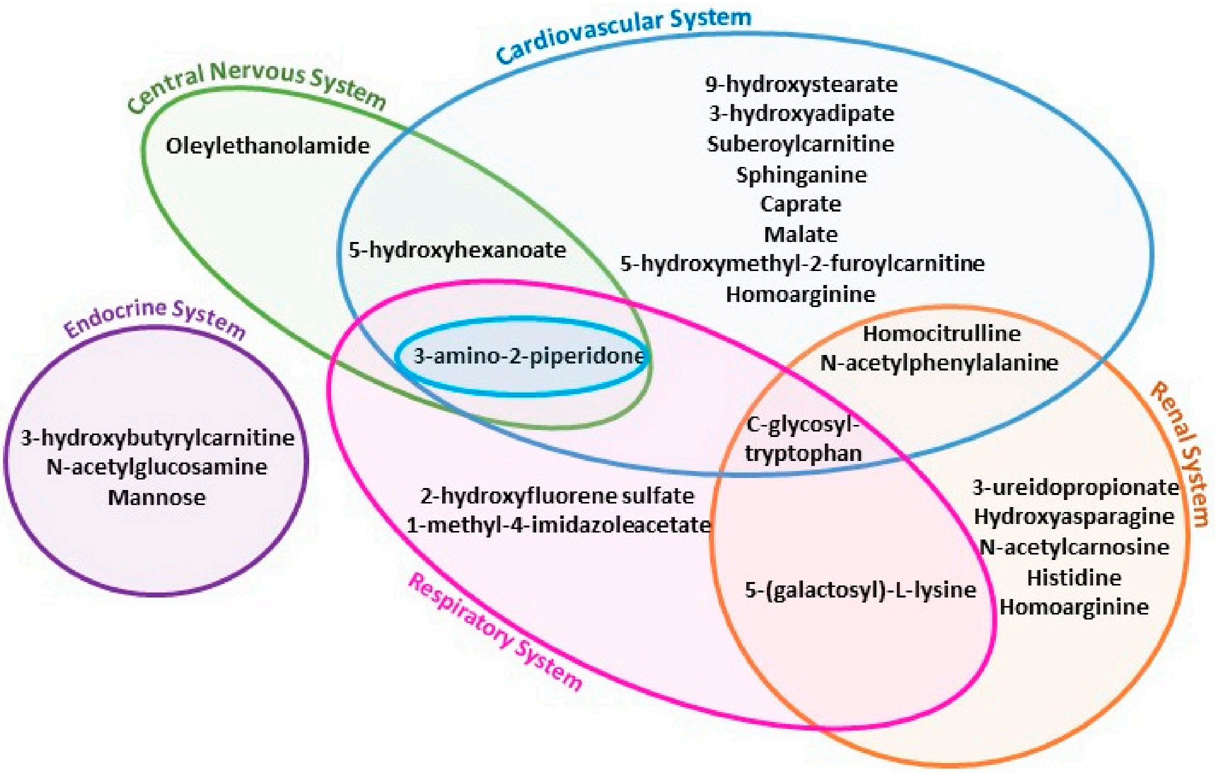

Figure S12. Veen diagram showing the body systems affected by the metabolites associated with the risk of mortality.
